# Discrete Event Simulation-Based Analysis and Optimization of Emergency Patient Scheduling Strategies

**DOI:** 10.3390/healthcare14010099

**Published:** 2025-12-31

**Authors:** Wei Lv, Runzhang Liu, Feiyi Yan, Yan Wang

**Affiliations:** 1 School of Economics and Management, Nanjing Tech University, Nanjing 210009, China; 202321118049@njtech.edu.cn (W.L.); 202321122019@njtech.edu.cn (R.L.); 2 School of Economics & Management, Chongqing Jiaotong University, Chongqing 400074, China; ywang2509@cqjtu.edu.cn; 3 Industrial Technology Research Institute, Chongqing Jiaotong University, Chongqing 400074, China

**Keywords:** emergency patient scheduling, discrete event simulation, dynamic scheduling strategies, resource allocation

## Abstract

**Background**: In the era of Health 4.0, Emergency Departments (EDs) face increasing crowding and complexity, necessitating smart management solutions to balance efficiency with equitable care. Effective scheduling is critical for optimizing patient throughput and mitigating congestion. **Methods**: This paper constructs a decision support framework using Discrete Event Simulation (DES) to evaluate three patient scheduling strategies, including the Initial-First policy, Alternating 1:1 policy and a Slack-Based dynamic policy. The simulation framework has been conducted using a standardized operational dataset representing typical ED dynamics. The threshold of SBP was optimized by a grid search method to guarantee an objective comparison. **Results**: The simulation results show that when adopting the optimized SBP policy, the mean waiting time was shortened by around 23.8%, thus meeting all triage service level targets. Also, it could be seen that Slack-Based dynamic policy was robust under different arrival rates and physician staffing levels. **Conclusions**: This proposed model can provide a real-time and dynamic solution for ED resource allocation, meeting the demand of modern smart hospitals management.

## 1. Introduction

Healthcare facilities are complex, resource-intensive systems, and their overall sustainability is a growing global concern. With the rapid development of Artificial Intelligence and the Internet of Medical Things, the focus of healthcare management has shifted from traditional administrative protocols to smart, algorithmic optimization of hospital operations. Specifically, leveraging digital tools and real-world operational data to enhance the efficiency of healthcare service delivery has become a central pillar of the “Smart Hospital” concept [1,2].

Emergency departments (EDs) are critical bottlenecks in healthcare systems that demand effective management strategies. EDs worldwide are under pressure from crowding due to rising patient volumes, increasing case complexity, and limited healthcare resources [3]. Such congestion results in prolonged waiting times, treatment delays, reduced patient satisfaction, and even higher in-hospital mortality rates [4,5]. As population aging and public health crises intensify these challenges, developing dynamic scheduling strategies to optimize patient flow has become a critical priority.

The foundation of patient scheduling begins with triage, which provides the institutional foundation for determining priority [6]. However, most hospitals still implement simple, static policies based on this triage, such as first-come–first-served or fixed priority rules [7,8]. While easy to implement, these static rules do not take advantage of the real-time operations now available in emergency departments. Theoretical queueing models demonstrate that simple priority queues can cause excessive delays for low-priority patients [9] or be suboptimal when downstream blocking occurs [10]. In practice, physicians’ operation-al decisions often deviate from these static rules during high congestion, suggesting their inadequacy [11].

Recognizing these limitations, a number of researchers have proposed more mature and flexible ways of managing limited resources with sophisticated dynamic scheduling approaches. Many papers attempted to develop job-shop scheduling models using genetic algorithm [12] or compose an opportunity constrained model [13] for ED-scheduling. Among these, slack-based strategies have emerged as a particularly promising dynamic alternative. This concept, initially proposed by Davenport et al. [14], uses temporal slack to enhance robustness. Subsequent applications in EDs have used deadline-aware control [15] and real-time algorithms according to residual slack [16], demonstrating that data-responsive dynamic scheduling outperforms conventional fixed-priority policies.

Methodologically, Discrete Event Simulation (DES) has become the indispensable tool for modeling and comparing the performance of these complex scheduling strategies [17,18]. Unlike analytical models, DES can explicitly represent the stochastic arrivals, heterogeneous patient paths, and resource competition inherent in ED operations [19]. Comprehensive reviews have confirmed that simulation-based methods are essential for assessing and improving ED performance [20]. Despite these advantages, a gap remains. Most prior studies examine isolated strategies rather than conducting comprehensive comparative evaluations of multiple representative strategies, such as static, alternating, and dynamic, within a single, unified simulation framework [21]. Moreover, systematic research applying these models to specific regional contexts, such as China’s four-level triage system, is still insufficient.

Recent studies have demonstrated the capability of analytical approaches to model increasingly complex systems, particularly through the use of Matrix-Analytic Methods as illustrated by Alexander Dudin and co-authors. These works successfully describe complex details such as correlated arrival processes and arrivals in groups [22,23]. Researchers have also improved these models to handle different types of customers, flexible entry rules [24], and systems with mixed service rules under limited resources [25]. These studies provide a strong foundation for understanding how these complex queueing networks work.

However, the emergency department system in this study has specific features that render exact mathematical solutions infeasible. First, the patient flow is not stable. Real-world data shows that arrival rates change significantly depending on the time and day, which is different from the stable conditions assumed by most analytical models. Second, it is very hard to model the competition for resources when the schedule depends on waiting times. To use these rules, the model must track exactly how long every patient has waited. This creates a huge number of system states, known as state space explosion. This makes calculation impossible. Therefore, discrete-event simulation is the best method.

To address these concerns, this work uses a discrete-event simulation (DES) model to evaluate and compare different scheduling approaches used for treating Level III and IV patients. In this model, three schedule design methods are utilized, namely the Initial-First Policy (IFP), the Alternating 1:1 Policy (ALT), and the Slack-Based Policy (SBP). The model represents patient-flow dynamics and event interactions using an event-driven queuing structure combined with discrete-event analytical procedures, enabling dynamic allocation of medical resources throughout the simulation process. A grid search approach was applied to find an optimal threshold for the delay tolerance parameter in the Slack-Based strategy, and the evaluation focused on average waiting time, total processing time, and service level compliance. In addition, the modeling could provide useful information for emergency care management on how to achieve timely as well as fair resource allocation.

There are two major innovations from this research, the first of which is establishing a unified Discrete Event Simulation (DES) model to evaluate medical resource allocation. This model evaluated and compared the performance of the Initial-First Policy, the Alternating 1:1 Policy, and the Slack-Based Policy using the Chinese four-tier triage system. The second innovation involved optimizing the Slack-Based Policy using a grid search algorithm to find the optimal threshold values and thereby greatly improved operations while providing a meaningful and intelligent decision making tool to hospitals in their management process.

The paper structure is as follows: In Section 2, the patient flow simulation modeling framework is described. Specifically, it covers the three-stage process of initial consultation, medical examination, and follow-up treatment. This section also elaborates on the mathematical implementation of the three scheduling strategies and defines the key performance metrics. In addition, this section proves the effectiveness of the simulation model. In Section 3, comparative analysis of waiting time, service level and resource usage are conducted based on different scheduling strategies. It also reports the parameter optimization results for the Slack-Based Policy. Moreover, this section performs sensitivity analysis to observe robustness under varying patient arrival rates and physician staffing levels. Finally, managerial implication and direction for future research is discussed in Section 4.

## 2. Materials and Methods

### 2.1. Problem Descriptions

**Figure 1:**
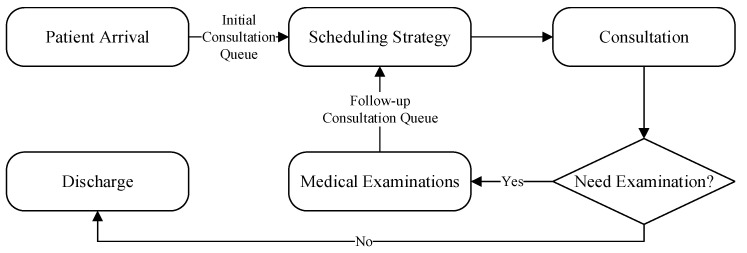
Flowchart of the Emergency Department System.

A study was conducted to analyze patient flow within an emergency department (ED) by measuring waiting times under different scheduling strategies. The patient-care process consists of three stages, namely the initial consultation, medical examination, and follow-up treatment, each constrained by the limited availability of physicians and diagnostic equipment, respectively. The overall configuration of the system is depicted in Figure 1.

#### 2.1.1. Patient Arrival and Triage

The patient journey begins upon arrival, which is modeled as a non-homogeneous Poisson process. The arrival rate $\lambda_{d,h}$ is dynamic, varying by both the day of the week (d) and the hour of the day (h) to reflect weekly periodic patterns.

Upon registration, each patient is triaged into one of four levels following the national emergency classification guideline. Level I (critical) and Level II (severe) patients receive immediate attention in resuscitation or intensive care units and are excluded from the present analysis. Level III and Level IV patients, representing moderate and low urgency cases, account for the majority of ED visits and constitute the primary population considered in this study. Their treatment process—comprising waiting, physician consultation, and potential diagnostic examinations—forms the basis of the scheduling model developed herein.

Let i=1,2,⋯,I denote the patient index, and $A_{arrive}^{t}$ represent the set of patients arriving at time t. Each newly arriving patient is assigned a triage level l∈{3,4}, corresponding to urgent and non-urgent cases, according to a probability mass function Pr(l).

#### 2.1.2. Consultation Process

Consultations are assigned to a designated group of physicians, who serve as the primary resources within the system. The number of doctors available $R^{t}$ varies across time periods according to their working shifts in the morning, afternoon, and night, and each shift is characterized by a distinct staffing level.

This physician pool has patients coming in from three different queues, including Level III initial patients $W_{init,3}^{t}$, Level IV initial patients $W_{init,4}^{t}$, and follow-up patients $W_{follow}^{t}$. The Consultation time is modeled by a truncated exponential distribution $T_{i}^{\left(k\right)}\sim TruncExp(\lambda_{k},\left[a_{k},b_{k}\right])$, with the distribution of time used differing between an initial consultation (k=1) and follow-up (k=2). These distributions are specified to take account for the different times required for these consultations according to the varying complexity of cases.

#### 2.1.3. Medical Examinations and Patient Flow

After the initial consultation, a patient is either discharged or, with a probability of $p_{exam}$, requires one or more medical examinations. The examination subsystem consists of J distinct modalities, such as laboratory tests, X-rays, and CT scans.

Each examination j is characterized by a processing time $\tau_{j}$, and a reporting delay $\delta_{j}$. Patients requiring multiple examinations are scheduled sequentially. The scheduling sequence is prioritized in descending order of reporting delay, such that $\delta_{j_{1}}\geq \delta_{j_{2}}\geq \cdots \geq \delta_{j_{m}}$, which ensures that tests with the longest reporting times are initiated first. An additional queueing delay $\rho_{j}$ may be incurred if all relevant devices are occupied. Once all examinations are completed and results are available at time $t_{i}^{exam}$, the patient joins the follow-up set $A_{follow}^{t}$ and enters the follow-up queue $W_{follow}^{t}$ to await their final consultation.

#### 2.1.4. The Scheduling Decision Mechanism

The core challenge addressed by this model is the dynamic allocation of available physicians $R^{t}$ among the three competing patient queues: $W_{init,3}^{t}$, $W_{init,4}^{t}$, and $W_{follow}^{t}$. A scheduling decision is triggered at any decision epoch t when a physician completes a service and becomes available, contingent upon at least one of the three patient queues being non-empty. If only one queue contains waiting patients, the first patient from that queue is immediately selected. However, when patients are waiting in multiple queues, the system must employ a priority rule to select the next patient. This selection is dictated by the specific scheduling strategy being evaluated, which can be based on criteria such as urgency level, queue length, or dynamic slack evaluation. This entire mechanism is designed to capture the dynamic allocation of medical resources and allows the model to illustrate the operational differences among the alternative policies.

### 2.2. Methodology

#### 2.2.1. Simulation Framework and Workflow

**Figure 2:**
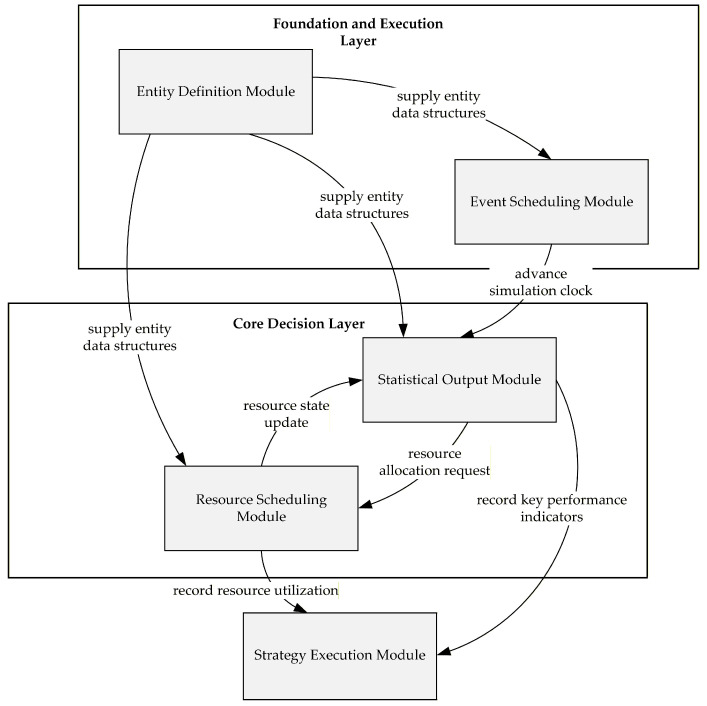
Modular architecture of the emergency department DES system.

A discrete-event simulation (DES) model was developed to evaluate alternative emergency patient scheduling strategies under stochastic arrivals, time-varying queue lengths, and resource competition. The DES framework is well suited for this context because patient arrivals, queue formation, resource contention, and service completion in the ED occur on an event-by-event basis. The simulation model was implemented in Python Version 3.13.3, utilizing a custom discrete-event engine built on priority queues to manage system dynamics. This event-driven framework maintains precise lists for patient arrivals, service completions, and re-source updates. The model adopts a modular architecture, as illustrated in Figure 2, consisting of five interdependent components.

The Entity Definition Module defines the data structures of patients and events. The Event Scheduling Module manages event generation and sequencing, advancing the simulation clock chronologically. The Resource Scheduling Module dynamically allocates physicians, consultation rooms, and diagnostic equipment according to availability. The Strategy Execution Module implements the alternative scheduling policies, including the Initial-First Strategy (IFP), the Alternating 1:1 strategy (ALT), and an optimized Slack-Based Strategy (SBP). It triggers decision-making whenever a resource becomes idle or a new patient arrives. The Statistical Output Module records and aggregates key performance indicators (KPIs), including waiting times, length of stay, and resource utilization. Through data exchange and event triggering, these modules form a cohesive logic that ensures a consistent and transparent representation of ED operations.

To ensure statistical robustness and minimize the impact of random fluctuations, 100 independent replications were conducted for each scheduling strategy, with each run representing one full operational week.

#### 2.2.2. Key Model Process and State Definitions

To ensure the model’s reproducibility and generality, this section defines the key variables, processes, and states implemented in the simulation. Table 1 summarizes the primary notation used in the model.

(1) Patient Arrival Process Implementation

The simulator generates patient arrival events according to the non-homogeneous Poisson process described in Section 2. At any given simulation time t, the corresponding day-of-week index d and hour-of-day index h are calculated via Equation (1):

(1) $$d=\left\lfloor \frac{t}{1440}\right\rfloor mod\;7,\;\;h=\left\lfloor \frac{t\;mod\;1440}{60}\right\rfloor$$

The empirical arrival rate $\lambda_{d,h}$ for that period is used to generate the number of arrivals within that hour, $N_{d,h}$, which follows a Poisson distribution:

(2) $$N_{d,h}\sim Poisson(\lambda_{d,h}),\;\;d=0,1,\cdots ,6;\;h=0,2,\cdots ,23$$

(2) Medical Examination Process Modeling

The simulator explicitly models the examination stage. For each patient i, the system assigns the need for examination j according to a probability $p_{j}$, represented by a Boolean indicator $x_{i,j}$:

(3) $$x_{i,j}\in \{0,1\}$$

where $x_{i,j}=1$ indicates patient i requires examination j. If the required device is occupied, the patient must wait, incurring an additional queueing delay $\rho_{j}$. As shown in Equation (4), the completion time of all examinations for patient i, $t_{i}^{exam}$, is defined as the latest completion time among all required examinations. This time accounts for the examination start time, processing duration ($\tau_{j}$), reporting delay ($\delta_{j}$), and queueing delay $\left(\rho_{j}\right)$:

(4) $$t_{i}^{exam}=\underset{k=1,\cdots ,m}{\max}\left(t_{i,j_{k}}^{start}+\tau_{j_{k}}+\delta_{j_{k}}+\rho_{jk}\right)$$

where $t_{i,j_{1}}^{start}=t_{i}^{exam\_start},\;t_{i,j_{k}}^{start}=t_{i,j_{k-1}}^{start}+\tau_{j_{k-1}},\;k=2,\cdots ,m$

(3) Queue Demand State Definition

Scheduling decisions rely on the real-time assessment of demand from the three primary queues. At any given decision epoch t, the system measures total demand D as the sum of the backlogged demand in the waiting queue W and the arrival of new patients A.

(5) $$D_{init,3}^{t}=W_{init,3}^{t}+\left|A_{init,3}^{t}\right|$$

(6) $$D_{init,4}^{t}=W_{init,4}^{t}+\left|A_{init,4}^{t}\right|$$

(7) $$D_{follow}^{t}=W_{follow}^{t}+\left|A_{follow}^{t}\right|$$

These demand state variables, identified as $D_{init,3}^{t}$ for total Level III initial demand, $D_{init,4}^{t}$ for total Level IV initial demand, and $D_{follow}^{t}$ for total follow-up demand, constitute the direct inputs for the scheduling strategies detailed in Section 2.2.3. As such, they form the foundation for the model’s decision-making logic.

#### 2.2.3. Scheduling Strategy Implementation

This section elaborates on specific algorithm realization methods used in the simulation model of the aforementioned three scheduling strategies. To handle resource contention consistently, the model applies a uniform rule across all strategies. Specifically, patients with equal priority are processed based on who arrived earliest, ensuring that the selection order is determined by arrival time rather than random selection.

(1) Initial-First Strategy

Under this policy, initial consultations always take precedence whenever their queues are non-empty, prioritized in the order Level III initial → Level IV initial → follow-up. The allocation of physician capacity at each decision epoch *t* is determined as follows:

(8) $$N_{init,3}^{t}=min(D_{init,3}^{t},R^{t})$$

(9) $$N_{init,4}^{t}=min(D_{init,4}^{t},R^{t}-N_{init,3}^{t})$$

(10) $$N_{follow}^{t}=min(D_{follow}^{t},R^{t}-N_{init,3}^{t}-N_{init,4}^{t})$$

Here, $N_{init,3}^{t}$, $N_{init,4}^{t}$, and $N_{follow}^{t}$ indicate the number of patients allocated to consultations at different time periods. Once a physician begins service, the service process will not be interrupted.

(2) Alternating 1:1 Strategy

This strategy attempts to balance resources between initial and follow-up patients. When both categories of queues are non-empty, physicians alternate between initial and follow-up patients at a 1:1 ratio. Within the initial consultation group, Level III patients are prioritized over Level IV patients.

At any given decision point, the number of initial consultations provided would be no more than half of the available physicians:

(11) $$N_{init,3}^{t}+N_{init,4}^{t}=min(D_{init,3}^{t}+D_{init,4}^{t},\left\lfloor \frac{R^{t}}{2}\right\rfloor )$$

The system first satisfies the Level III initial demand, then allocates the remaining initial-patient quota to Level IV:

(12) $$N_{init,3}^{t}=min(D_{init,3}^{t},\left\lfloor \frac{R^{t}}{2}\right\rfloor ),\;\;N_{init,4}^{t}=min(D_{init,4}^{t},\left\lfloor \frac{R^{t}}{2}\right\rfloor -N_{init,3}^{t})$$

Finally, all remaining physician capacity is allocated to the follow-up queue:

(13) $$N_{follow}^{t}=min(D_{follow}^{t},R^{t}-N_{init,3}^{t}-N_{init,4}^{t})$$

When a queue contains fewer patients than what it was allocated, the remainder of the capacity is dynamically reassigned to other queues to maximize the use of resources.

(3) Slack-Based Strategy

This strategy introduces a time-based urgency, setting waiting time thresholds $T_{3}$ and $T_{4}$ for Level III and Level IV initial patients, respectively. Slack tolerance parameters $k_{1}$ and $k_{2}$ define how far in advance of these thresholds a patient is considered “urgent.”

A patient i is flagged as “urgent” at time t if their waiting time $w_{i}(t)$ satisfies the condition in Equation (14):

(14) $$w_{i}(t)\geq T_{l}-k_{l}$$

This defines two dynamic “urgent sets” at time t:

(15) $$U_{3}^{t}(k_{1})=\{i:w_{i}(t)\geq T_{3}-k_{1}\},U_{4}^{t}(k_{2})=\{i:w_{i}(t)\geq T_{4}-k_{2}\}$$

At decision epoch t, the system applies the following priority hierarchy:

(16) $$U_{3}^{t}(k_{1})>U_{4}^{t}(k_{2})>F^{t}>H_{init}^{t}$$

where $F^{t}$ indicates the follow-up queue while $H_{init}^{t}$ refers to the remaining non-urgent initial patients. For each group, Level III has higher priority than Level IV, which assigns the physicians in turn according to the sequence of these priorities:

(17) $$n_{3}=min(\left|U_{3}^{t}\right|,R^{t})$$

(18) $$n_{4}=min(\left|U_{4}^{t}\right|,R^{t}-n_{3})$$

(19) $$n_{f}=min(\left|F^{t}\right|,R^{t}-n_{3}-n_{4})$$

(20) $$n_{r}=min(\left|H_{init}^{t}\right|,R^{t}-n_{3}-n_{4}-n_{f})$$

At the end of each time step, patients who have left the queue during that step are removed from it, while those remaining are carried over to the next time step:

(21) $$W_{init,3}^{t+1}=W_{init,3}^{t}+D_{init,3}^{t}-n_{3}$$

(22) $$W_{init,4}^{t+1}=W_{init,4}^{t}+D_{init,4}^{t}-n_{4}$$

(23) $$W_{follow}^{t+1}=W_{follow}^{t}+D_{follow}^{t}-n_{f}$$

(4) Performance Metrics

In order to compare the advantages of different solutions in the simulation process, it is necessary to monitor key performance indicators:

(1) Average Waiting Time:
  - Average waiting times for level l initial patients, ${\bar{W}}_{init,l}$
    (24) $${\bar{W}}_{init,l}=\frac{1}{\left|S_{init,l}\right|}\sum_{i\in S_{init,l}}w_{l}$$
  - Average waiting times for follow-up patients, ${\bar{W}}_{follow}$
    (25) $${\bar{W}}_{follow}=\frac{1}{\left|S_{follow}\right|}\sum_{i\in S_{follow}}w_{i}$$
  - Overall average waiting time across all patients, ${\bar{W}}_{total}$
    (26) $${\bar{W}}_{total}=\frac{\sum_{i\in S_{init,3}⋃S_{init,4}{⋃S}_{follow}}w_{i}}{\left|S_{init,3}\right|+\left|S_{init,4}\right|+\left|S_{follow}\right|}$$
    where $S_{init,3}$, $S_{init,4}$ and $S_{follow}$ denote the sets of patients who completed their consultations during the simulation period.
(2) Overtime Ratio/Service Level:
  - The proportion of patients of level l patients whose waiting times exceeds the clinical time $T_{l}$, denoted as $\Delta_{l}$
    (27) $$\Delta_{l}=\frac{\sum_{i\in S_{init,}l}{1(w}_{i}>T_{l})}{\left|S_{init,l}\right|},\;\;for\;l\epsilon \{3,4\}$$
    where ${1(w}_{i}>T_{l})$ is the indicator function.
  - Service level ${SL}_{l}$, defined as the complement of the overtime ratio $1-\Delta_{l}$
    (28) $${SL}_{l}=1-\Delta_{l}=\frac{\sum_{i\in S_{init,}l}{1(w}_{i}<T_{l})}{\left|S_{init,l}\right|},\;\;for\;l\epsilon \{3,4\}$$
(3) Resource Utilization
  - Physician utilization $U_{doc}$: the ratio of busy time to total available time for physicians
    (29) $$U_{doc}=\frac{\sum_{t=1}^{T}\sum_{s}N_{severd}^{t,s}}{\sum_{t=1}^{T}R^{t}}$$
    where $N_{severd}^{t,s}$ be the total number of consultations completed during shift s on day t.
  - Resource utilization $U_{j}$: the ratio of busy time to total available time for devices j
    (30) $$U_{j}=\frac{\sum_{t=1}^{T}min(D_{j}^{t},C_{j})}{\sum_{t=1}^{T}C_{j}},\;j=1,2,\cdots ,J$$

#### 2.2.4. Model Validation

To validate the model, the simulation follows the national four-tier triage system of China. Level I and Level II patients are critical and are sent directly to resuscitation units. Therefore, they are excluded from the general consultation queues. Instead, the simulation focuses on Level III and Level IV patients. This approach reflects real-world operations where critical patients receive immediate care. By focusing on these moderate and non-urgent cases, the model accurately represents the resource competition in the emergency department.

Furthermore, the operational dynamics are modeled to faithfully reproduce real-world complexity. The simulation traces the complete patient journey by integrating triage, initial consultation, diagnostic procedures, and follow-up consultation. Within this process, patients arrive in varying volumes throughout the day and week, creating realistic demand fluctuations. The system utilizes an event-driven mechanism where physicians are dynamically allocated to patients from the queue based on the active schedule. Finally, the patient arrival patterns were calibrated against historical data to ensure that the simulated load aligns with actual operations, thereby guaranteeing flow conservation.

#### 2.2.5. Justification for the Simulation Approach

This study uses Discrete-Event Simulation to evaluate the proposed scheduling strategies. Although analytical methods like Matrix-Analytic Methods work well for some systems, the specific features of our model make simulation the best choice.

The choice of simulation is justified by three key complexities that preclude exact analytical solutions. First, the system is highly non-stationary. As shown in Table 2, patient arrival rates vary significantly by hour and day, and staffing levels fluctuate across shifts, which makes steady-state analytical frameworks inapplicable. Second, the service process violates standard Markovian assumptions. The use of truncated exponential distributions and fixed deterministic durations for equipment checks is incompatible with the memoryless property required by most analytical models. Finally, the simulation explicitly captures the history-dependent Slack-Based Policy. Analytically modeling this strategy requires tracking the exact waiting history of every individual patient as a state variable. This leads to state space explosion, where the massive increase in system states renders exact mathematical derivation computationally intractable. Therefore, Discrete-Event Simulation provides a more robust and feasible platform for this study.

## 3. Results

### 3.1. Simulation Setup

The simulation model is parameterized using a standardized operational dataset derived from the actual records of a Grade-A tertiary hospital in China. This dataset captures critical real-world ED dynamics, including the distribution of heterogeneous patient arrivals, stochastic service times, and resource constraints. Integrating these empirical factors ensures the model’s validity and reliability for evaluating scheduling strategies

Based on this dataset, patient inflow was modeled as a piecewise-constant, non-homogeneous Poisson process with a weekly cycle to replicate the temporal dynamics of emergency department arrivals. The specific hourly arrival rates are presented in Table 2. In alignment with the case study’s observed proportions, Level III and Level IV patients accounted for 25% and 75% of arrivals, respectively. The corresponding target waiting times were set at 30 min for Level III and 120 min for Level IV.

For these arriving patients, service durations were defined. Initial and follow-up consultations followed truncated exponential distributions with means of 9 and 15 min, respectively. The truncation bounds were 5–15 min for initial consultation and 5–25 min for follow-up consultation. Examination times were set at 1.19 min for laboratory tests, 6.58 min for B-ultrasound, 3.99 min for X-ray, and 2.45 min for CT. Report availability delays were assumed to be 20 min for laboratory tests, immediate for B-ultrasound, and 30 min for both X-ray and CT. Among initial patients, 60% required at least one diagnostic test, with independent probabilities of undergoing laboratory, B-ultrasound, X-ray, and CT set at 92%, 22%, 29%, and 55%, respectively.

To manage these service processes, the emergency department operated in three shifts per day, namely 07:00–15:00 with 5 physicians, 15:00–22:00 with 5 physicians, and 22:00–07:00 with 3 physicians, reflecting typical staffing levels observed in the hospital.

### 3.2. Optimization of Slack-Based Strategy

Unlike the static Initial-First and Alternating 1:1 policies, the performance of the Slack-Based strategy is directly contingent upon its delay tolerance parameters, $k_{1}$ and $k_{2}$. Therefore, before conducting a comprehensive comparison against the other strategies, this section first optimizes these parameters.

The goal is to find the optimal parameter pair by minimizing the overall average patient waiting time, ${\bar{W}}_{total}(k_{1},k_{2})$, while adhering to minimum service-level (SL) constraints. This optimization problem is formally defined as:

(31) $$\underset{k_{1},k_{2}}{\min}{\bar{W}}_{total}(k_{1},k_{2})$$

subject to:

(32) $${SL}_{3}\geq {SL}_{3}^{min},\;\;{SL}_{4}\geq {SL}_{4}^{min}$$

To resolve this, we apply a two-stage iterative grid search method that derives the best possible setting for parameters. Based on evaluating the simulation result under a large number of thresholds, we get the parameter thresholds that can be applied in the next trip.

In the first phase, a coarse search with a step size of 1 was conducted to determine the neighborhood of the optimal solution. Table 3 presents the five best-performing parameter combinations from this initial search, ranked by average waiting time. The analysis identified the parameter pair of 13 and 2 as the preliminary optimum, achieving a mean waiting time of 36.38 min. To further refine this result, a fine-grained search was subsequently performed using a step size of 0.1 within the neighborhood of the preliminary optimum, specifically covering the range from 12 to 14 and 1 to 3. Table 4 lists the top outcomes from this second phase. This precise optimization revealed that the parameter pair of 13.1 and 2.1 is the global effective combination, further reducing the lowest mean waiting time to 35.25 min.

Notably, this final configuration demonstrates high reliability, with a standard deviation of 6.10 min and a 95% confidence interval ranging from 34.06 to 36.45 min. This robust statistical profile confirms the stability of the selected parameters and validates their suitability for operational implementation.

### 3.3. Comparative Analysis of Scheduling Strategies

The performance of the emergency department under the three scheduling strategies was evaluated using the metrics from Section 2.2.3. The following sections compare the Initial-First, Alternating 1:1, and the optimized Slack-Based ($k_{1}=13.1$, $k_{2}=2.1$) strategies in terms of average waiting time, service level, and resource utilization. To ensure statistical robustness, each strategy was assessed over 100 independent replication runs.

#### 3.3.1. Average Waiting Time Comparison

**Figure 3:**
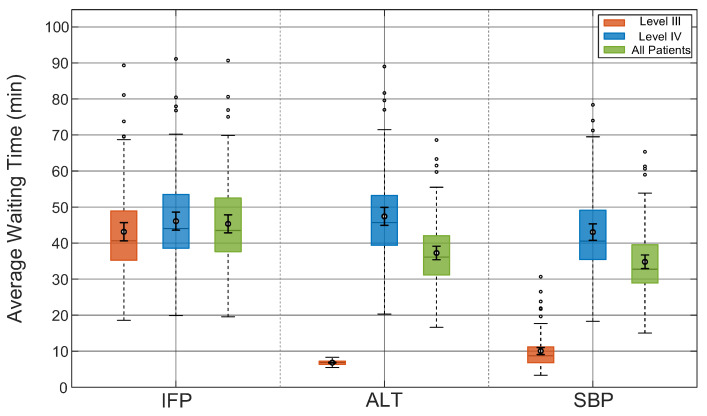
Comparison of average waiting time under three scheduling strategies.

As shown in Figure 3, the average waiting times of Level III patients, Level IV patients, and all patients were compared under the three scheduling strategies.

The results highlight clear performance differences among the three scheduling strategies. The longest mean waiting times resulted in the Initial-First strategy, 46.26 min overall. This approach was particularly ineffective for urgent Level III patients who had to wait about 44.14 min, indicating that the strategy failed to adequately prioritize urgent cases. The 1:1 Alternating Strategy shortened Level III’s average wait time from 44.14 to 6.82 min, and achieved an overall average of 37.88 min, approaching the minimum possible value. However, this improvement was accompanied by a substantial increase in the waiting times of the already disadvantaged Level IV patients.

The Slack-based strategy achieved the best performance, with an overall combined waiting time of 35.25 min. Although the Level III waiting time, at 10.48 min, was longer than that under the Alternating strategy, it achieved the shortest delay for Level IV patients at 43.89 min. These results demonstrate that the Slack-based strategy achieved an excellent balance between speed and fairness. Regarding the data distribution, although outliers as indicated by the circles in Figure 3 were observed, the SBP strategy showed a more stable performance. Beyond the mean values, the 95% confidence intervals reinforce the robustness of these findings. The CI for SBP [34.06, 36.45] is notably narrower than that of IFP [43.76, 48.76], and there is no overlap between them, demonstrating a statistically significant improvement over the baseline strategy.

To test whether the differences in the performance of the model are statistically significant or only phenomena with trivial practical effects, we employ a complete set of statistical tests to measure both their statistical and practical significance between the three strategies. Paired *t*-test, Bonferroni correction for multiple comparisons, and Cohen’s d for effect size were utilized in our research.

(1) Assessment of Statistical Significance

Paired *t*-tests were performed on the mean waiting times of the three strategies under identical arrival seeds to detect systematic differences. To rigorously control the family-wise error rate across the three pairwise comparisons, a Bonferroni correction was applied. This adjusted the standard significance level (α=0.05) to a stricter threshold calculated as ($\alpha_{adj}=0.0167$).

The results, summarized in Table 5, show that all comparisons yielded extremely large t-statistics and negligible *p*-values. Notably, all *p*-values were far below the adjusted threshold of 0.0167, indicating that the observed differences were attributed to inherent performance gaps between strategies rather than random fluctuations. The direction of the effect was unambiguous: SBP consistently generated the shortest waiting time, followed by ALT, with IFP incurring the longest delay.

(2) Assessment of Practical Significance

To assess the practical magnitude of these differences beyond mere statistical significance, Cohen’s d was calculated for each pairwise comparison. According to conventional benchmarks, values of 0.2, 0.5, and 0.8 indicate small, medium, and large effects, respectively, while values exceeding 1.2 represent a very large effect.

It can be seen from Table 6 that the three comparisons produced Cohen’s d greater than 1.5 and the difference between IFP and SBP had a huge effect size of 2.82. Thus, the improvement of SBP must possess statistical strength as well as practical benefits to clinical workflow.

#### 3.3.2. Delay Incidence and Service Level Analysis

Table 7 details the incidence of delays and the resulting service levels. A delay is defined as a waiting time exceeding 30 min for Level III or 120 min for Level IV patients.

The simulation data exposes the hidden cost of the ALT strategy. While it prioritizes urgent patients, it causes a significant delay rate of 10.71% for Level IV patients, reducing their service level to only 89.29%. This unacceptable drop in service quality highlights the risk of rigid alternating rules. In contrast, SBP achieves a 0.00% delay rate for all patients, ensuring maximum service levels. This confirms that its dynamic prioritization effectively mitigates non-urgent patient accumulation, balancing urgency and fairness more robustly than the ALT strategy.

#### 3.3.3. Resource Utilization Analysis

Table 8 summarizes physician utilization under the three scheduling strategies, with all data representing weekly averages over a seven-day period. The results indicate that physician utilization remained consistently high, approximately 85% under all strategies. This consistency suggests that medical resources were effectively used across different scheduling policies. Operational stability can be demonstrated through dispersion statistics, as uniform standard deviations ranging between 2.06% to 2.08% shows that the operational loads are fairly constant with very little change. And the range in utilization from 79% to 91% indicates high demand which has been persistent over time. In addition, the similar distributions around the mean and median show that the need for frequent high utilizations is a constant state, rather than an unusual occurrence.

Table 9 presents the utilization of examination equipment under the three scheduling strategies, with all values based on weekly averages. Notably, the utilization rates remain identical across strategies. This outcome reflects a structural feature of the simulation model, where each patient type has a fixed probability of requiring specific diagnostic tests. Since the scheduling strategies only influence the order in which patients are treated, without affecting the overall volume of examinations, the total workload on each device remains unchanged.

The data reveal that all diagnostic equipment is significantly underutilized, with utilization rates ranging from 14.36% for laboratory services to 17.64% for CT and B-ultrasound. Further evidence is given by the low standard deviations lying between 0.40% and 1.01%, which means there is hardly any variation in day-to-day operation. The low margins also indicate that the need for resource for diagnostics does not vary significantly during one simulation. This combination of low utilization and high stability stands in sharp contrast to the approximately 85% physician utilization observed in Table 5, indicating that physician availability, rather than equipment, is the primary bottleneck in the system. This study implies that procuring extra diagnostic equipment may yield fewer benefits than initially expected. A more effective approach to increase patient throughput and reducing waiting time for patients with limited resources is to streamline the management of physician schedules.

### 3.4. Sensitivity Analysis

A sensitivity analysis was undertaken to assess whether the optimized Slack-Based model retains its advantages under different operating conditions. Two parameters were manipulated: the patient arrival rate and the number of physicians. The arrival rate, being a significant source of demand variability, was tested to ensure the optimization technique worked at different levels of demand; the number of physicians, on the other hand, represents an example of organizational constraints, affecting how scaling occurs and resource utilization.

#### 3.4.1. Impact of Patient Arrival Rate

**Figure 4:**
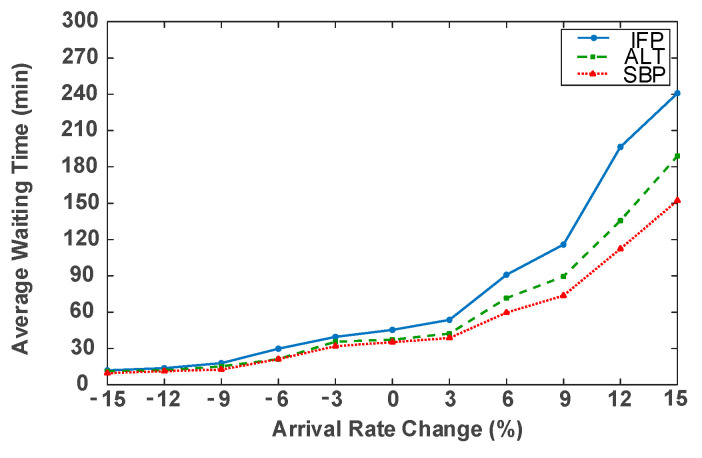
Impacts of arrival rate change on average waiting time.

To examine robustness under varying demand conditions, the patient arrival rate was systematically adjusted within ±15% of the baseline in 3% increments. A series of low- and high-load scenarios were simulated, and all three scheduling strategies were re-evaluated across these conditions. Figure 4 presents the corresponding changes in overall average waiting time.

It can be observed from Figure 4, under low-load situation, the average waiting time of all schemes are basically in line with each other and the difference is marginal. Furthermore, compared with other two schemes, the slack-based strategy exhibits slightly better performance, indicating that it allocates resource more effectively under lower loads. As the arrival rate increases and the system moves from a low-load to a busier state, the differences in performance among the strategies become more noticeable. The waiting times under the Initial-first and Alternating 1:1 strategies grow quickly, while the Slack-Based method consistently outperforms the others.

#### 3.4.2. Impact of Physician Staffing

**Figure 5:**
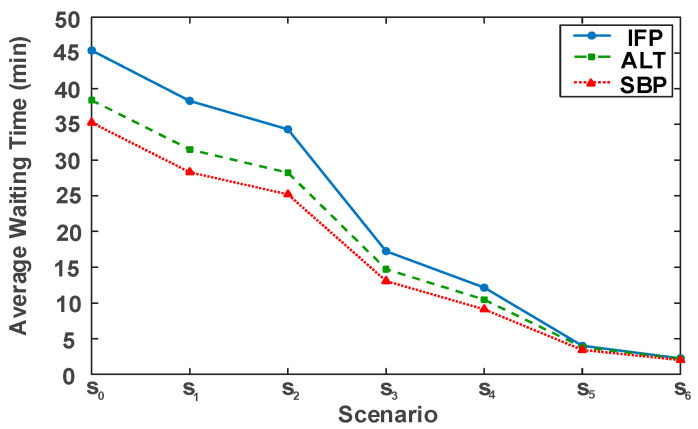
Impacts of physician capacity change on average waiting time.

To examine the scaling capability of the model, we varied the staffing level of physicians in the simulation. Seven scenarios (S0–S6) were defined, starting with the baseline scenario (S0) and gradually adding more physicians, as listed in Table 10. The simulation was rerun using the three scheduling methods for these seven cases. These results are shown in Figure 5.

Figure 5 shows that increasing physician staffing can greatly reduce average waiting times across all strategies; however, the gaps among strategies vary significantly with resources. Specifically, when resources are scarce (e.g., S0–S3), the SBP strategy performs best. Compared to IFP and ALT, it can make maximum use of limited capacity to substantially reduce queue accumulation under the condition of constrained resources. As more resources become available (e.g., S4–S6), the average waiting times for all strategies continue to decline, and the performance gaps progressively narrow. This trend indicates diminishing marginal returns from adding staff, as system congestion is broadly alleviated. Throughout this phase, the SBP continues to outperform and maintain its position as the top-performing strategy.

#### 3.4.3. Impact of Service-Time Distributions

To examine the robustness of the simulation results under different service-time distributions, the truncated exponential distributions originally assumed for consultation times are replaced by truncated lognormal distributions, while all other model settings are kept unchanged.

In the baseline model, consultation times for initial and follow-up visits follow truncated exponential distributions with mean values of 9 and 15 min, truncated to [5,15] and [5,25] minutes, respectively. In the robustness analysis, truncated lognormal distributions are adopted with parameters calibrated to preserve the same expected service times. Specifically, for initial consultations,

$$\mu =ln(9)-\frac{1}{2}\sigma^{2},\;\sigma =0.3$$

for follow-up consultations,

$$\mu =ln(15)-\frac{1}{2}\sigma^{2},\;\sigma =0.3$$

The simulation results reveal that substituting the distribution leads to a general increase in waiting times. Under the truncated lognormal assumption, the average waiting times for the three strategies rose to 59.75, 53.04, and 51.56 min, respectively, compared to 46.26, 37.88, and 35.25 min in the baseline model. This increase is primarily driven by the heavier right tail and greater variability inherent in the lognormal distribution, which are known to exacerbate congestion in queueing systems.

Despite the increase in absolute waiting times, the relative performance ranking of the three scheduling strategies remains unchanged. The Slack-Based Policy (SBP) continues to outperform the others, followed by the Alternating Policy (ALT). This consistency indicates that the comparative advantages of the proposed strategies are not driven by a specific choice of service-time distribution, but rather reflect their intrinsic structural differences. Therefore, the study’s main conclusions are robust to reasonable variations in service-time distributions.

## 4. Conclusions

This study developed a discrete-event simulation (DES) model to examine various scheduling strategies concerning Level III and Level IV patients in the Emergency Department. As a core finding, the dynamically optimized Slack-Based policy outperformed the commonly used static rule-based strategies. Specifically, the SBP reduced the average waiting time for both groups of patients from 46.26 to 35.25 min, and eliminated all service-level delays.

The results show important managerial implications. First, the sensitivity analysis demonstrates the system bottleneck resource is physician capacity and not diagnostic equipment, thereby suggesting that increasing the number of physicians available at the hospital can be done instead of spending more money on acquiring extra equipment. Second, tuning the SBP parameters through grid search provides a transparent and interpretable form of prescriptive analytics. This method identifies suitable parameter settings through an open evaluation process, making the resulting decision rules easier for clinicians to understand and apply.

However, several limitations of this paper are observed and possible further directions are proposed for following works.

First, regarding the data detail, our model simplifies the link between patient types and examination choices because our dataset is aggregated. We did not model the exact link between every symptom and exam. However, this does not affect the main results. As shown in Table 9, the diagnostic equipment is not busy, with utilization rates under 20%. This confirms that the bottleneck is the doctors, not the equipment. Therefore, the ranking of the strategies remains valid. In the future, we aim to use more detailed patient data to capture these specific connections.

Second, regarding validation, we used real-world arrival patterns to build the model. However, we could not validate the exact waiting times against historical data because that specific data was not available. Therefore, the main goal of this study is to compare the strategies against each other, rather than to predict absolute values. Even without perfect validation, the model correctly identifies the system bottlenecks and how the strategies perform relative to each other. Future studies can use complete Electronic Health Records for stricter validation.

Third, regarding the method, we currently tuning the parameters with grid search. This method is fine for simple problems and it’s clear and straightforward. However, it becomes slow and hard to use when there are lots of decision variables. With large models that incorporate many more departments and resources, the problem will become much more complicated. To address this, we plan to use Deep Reinforcement Learning in the future. This advanced technique can be able to tackle large scale problems and balance between efficiency, fairness and others different aspects like that.

## Figures and Tables

**Table 1:** Key variables used in the model.

Symbol	Description
t	Simulation time epoch
T	Total simulated minutes
$N^{t}$	Cumulative ED arrivals by time t
d	Day-of-week index
h	Hour-of-day index
$\lambda_{d,h}$	Arrival rate for day d, hour h
$N_{d,h}$	arrivals in hour h of day d
i	Patient index
$A_{arrive}^{t}$	New arrivals at epoch t
l	Acuity level
Pr(l)	Probability mass of level l
$x_{i,j}$	Exam- j required by patient i
$p_{j}$	Probability exam j is required
$W_{j}^{t}$	Backlog of device j at start of t
$D_{j}^{t}$	Total demand for device j at time t
$\tau_{j}$	Exam duration on device j
$\delta_{j}$	Result-report delay for device j
$\rho_{j}$	Extra delay if j busy
$t_{i,j_{k}}^{start}$	Start of i’s k-th exam on j
$t_{i}^{exam}$	Time patient i finishes exams
$A_{follow}^{t}$	Patients awaiting follow-up at t
k	1 = initial, 2 = follow-up
$T_{i}^{\left(k\right)}$	Type-k consult time for patient i
$\lambda_{k}$	Rate parameter
$\left[a_{k},b_{k}\right]$	Duration bounds
$D_{init,3}^{t}$	Total urgent-initial demand at t
$D_{init,4}^{t}$	Total low-urgent-initial demand at t
$D_{follow}^{t}$	Total follow-up demand at t
$W_{init,3}^{t}$	Urgent initial queue at t
$W_{init,4}^{t}$	Less-urgent initial queue at t
$W_{follow}^{t}$	Follow-up queue at t
$A^{t}$	Newly arrived patients at t
$R^{t}$	Available physicians at t
$k_{1},k_{2}$	Early-urgent thresholds
$T_{l}$	time limit

**Table 2:** The average patient arrival rate per hour.

Hours	Mon.	Tue.	Wed.	Thur.	Fri.	Sat.	Sun.
0–1	7.84	7.5	7.51	7.49	7.49	7.13	7.12
1–2	5.79	5.37	5.38	5.35	5.36	5.07	5.06
2–3	4.94	4.5	4.51	4.5	4.49	4.31	4.32
3–4	3.7	3.33	3.34	3.3	3.34	3.11	3.12
4–5	2.19	1.83	1.81	1.8	1.82	1.68	1.68
5–6	4.33	3.58	3.59	3.54	3.55	3.21	3.19
6–7	6.78	6.39	6.4	6.37	6.36	6.08	6.07
7–8	8.83	8.23	8.17	8.18	8.25	7.98	7.97
8–9	15.23	14.95	14.93	14.9	14.9	14.49	14.48
9–10	21.59	21.26	21.24	21.23	21.27	20.64	20.61
10–11	21.71	21.44	21.43	21.45	21.43	20.65	20.64
11–12	17.53	17.11	17.12	17.11	17.1	16.59	16.51
12–13	15.55	15.18	15.17	15.18	15.17	14.73	14.72
13–14	18.26	17.93	17.94	17.92	17.92	17.43	17.41
14–15	20.87	20.76	20.77	20.75	20.77	19.97	19.96
15–16	18.28	18.14	18.15	18.13	18.13	17.33	17.35
16–17	17.31	17.14	17.15	17.13	17.14	16.47	16.45
17–18	16.28	16.1	16.11	16.08	16.1	15.38	15.39
18–19	16.87	16.46	16.44	16.43	16.44	15.97	15.98
19–20	22.61	22.47	22.46	22.43	22.44	21.64	21.63
20–21	23.05	22.81	22.8	22.79	22.79	22.44	22.43
21–22	17.53	17.23	17.24	17.21	17.22	16.75	16.74
22–23	12.49	12.31	12.3	12.29	12.29	11.83	11.82
23–0	7.52	7.23	7.21	7.22	7.24	7.03	7.02

**Table 3:** Top performing parameter combinations from the coarse grid search (Step size = 1.0).

Rank	Parameter Pair ($k_{1},k_{2}$)	Average Waiting Time (min)	Standard Deviation	95% Confidence Interval
1	13, 2	36.38	11.27	[33.25, 39.50]
2	15, 11	36.90	11.51	[33.71, 40.09]
3	14, 0	36.96	12.13	[33.59, 40.32]
4	26, 2	37.10	11.40	[33.93, 40.26]
5	10, 40	37.69	11.53	[34.50, 40.88]

**Table 4:** Top performing parameter combinations from the fine grid search (Step size = 0.1).

Rank	Parameter Pair ($k_{1},k_{2}$)	Average Waiting Time (min)	Standard Deviation	95% Confidence Interval
1	13.1, 2.1	35.25	6.10	[34.06, 36.45]
2	13.4, 2.1	35.28	6.16	[34.08, 36.49]
3	12.8, 1.7	35.30	5.83	[34.11, 36.44]
4	13.1, 1.6	35.30	6.10	[34.11, 36.49]
5	13.3, 2.1	35.31	6.10	[34.15, 36.50]

**Table 5:** Paired *t*-test Results and Statistical Significance.

Comparison	Mean Diff.	*t*-Value	*p*-Value	Significant
IFP vs. ALT	8.1000	20.099377	$1.015178\times 10^{-36}$	Yes
IFP vs. SBP	10.5521	28.244962	$3.580432\times 10^{-49}$	Yes
ALT vs. SBP	2.4521	15.436824	$4.208457\times 10^{-28}$	Yes

**Table 6:** Effect Sizes (Cohen’s d) for Comparisons of Three Scheduling Strategies.

Comparison	Cohen’s d	Interpretation	Practical Meaning
IFP vs. ALT	2.0099	Very large effect	Substantial reduction in waiting time
IFP vs. SBP	2.8245	Huge effect	Dramatic efficiency gain
ALT vs. SBP	1.5437	Very large effect	Further noticeable improvement

**Table 7:** Delay incidence and service level compliance.

Strategy	Patient Level	Delay Rate (Mean ± SD)	Service Level (Mean ± SD)
IFP	Level III	0.00±0.00%	100.00±0.00%
	Level IV	0.00±0.00%	100.00±0.00%
ALT	Level III	1.92±1.01%	98.08±1.01%
	Level IV	10.71±5.44%	89.29±5.44%
SBP	Level III	0.00±0.00%	100.00±0.00%
	Level IV	0.00±0.00%	100.00±0.00%

**Table 8:** Physician utilization statistics.

Strategy	Mean Utilization	Standard Deviation	Min	Max	Median
IFP	85.43%	2.07%	79.63%	91.08%	85.38%
ALT	85.41%	2.08%	79.39%	91.11%	85.41%
SBP	84.98%	2.06%	79.06%	90.50%	84.80%

**Table 9:** Examination Equipment Utilization across All Scheduling Strategies.

Equipment	Number of Exams	Average Duration per Exam (min)	Total Duration (min)	Available Time (min)	Utilization	Standard Deviation
XRAY	389	3.99	1552.11	10,080	15.20%	0.78%
CT	715	2.45	1751.75	10,080	17.64%	0.71%
LABORATORY	1228	1.19	1461.32	10,080	14.36%	0.40%
ULTRASOUND	266	6.58	1750.28	10,080	19.03%	1.01%

Note: Equipment utilization rates were statistically consistent across all three strategies (IFP, ALT, and SBP), as diagnostic demand is determined by patient arrival characteristics rather than physician scheduling logic.

**Table 10:** Physician shift configurations for sensitivity analysis.

Scenario	Morning Shift	Afternoon Shift	Night Shift	Configuration (M–A–N)
$S_{0}$	5	5	3	(5, 5, 3)
$S_{1}$	5	5	4	(5, 5, 4)
$S_{2}$	5	5	5	(5, 5, 5)
$S_{3}$	5	6	5	(5, 6, 5)
$S_{4}$	5	7	5	(5, 7, 5)
$S_{5}$	6	7	5	(6, 7, 5)
$S_{6}$	7	7	5	(7, 7, 5)

## Data Availability

The data presented in this study are available on request from the corresponding author. (The organizers only release de-identified data to the participants).

## Abbreviations

The following abbreviations are used in this manuscript:

Eds	Emergency Departments
DES	Discrete Event Simulation
IFP	Initial-First Strategy
ALT	Alternating 1:1 Strategy
SBP	Slack-Based Strategy
